# Baicalin induces cellular senescence in human colon cancer cells via upregulation of DEPP and the activation of Ras/Raf/MEK/ERK signaling

**DOI:** 10.1038/s41419-017-0223-0

**Published:** 2018-02-13

**Authors:** Zhou Wang, Lingman Ma, Mengqi Su, Yiran Zhou, Ke Mao, Chengqin Li, Guangyong Peng, Changlin Zhou, Baiyong Shen, Jie Dou

**Affiliations:** 1 State Key Laboratory of Natural Medicines, School of Life Science and Technology, China Pharmaceutical University, Nanjing, 210029 China; 2 Department of General Surgery, Ruijin Hospital, Research Institute of Pancreatic Diseases, School of Medicine, Shanghai JiaoTong University, Shanghai, 200025 China; 3 Division of Infectious Diseases, Allergy & Immunology and Department of Internal Medicine, Saint Louis University School of Medicine, Saint Louis, MO 63104 USA

## Abstract

Baicalin is a natural flavonoid glycoside which has potent anti-tumor and antioxidant activity in cancer cells. In the present study, we found that baicalin treatment significantly induced senescence in colon cancer cells. Furthermore, baicalin upregulated the expression of decidual protein induced by progesterone (DEPP) in HCT116 colon cancer cells, which accompanied with the activation of Ras/Raf/MEK/ERK and p16^INK4A^/Rb signaling pathways. Meanwhile, these phenomena also appeared under the anti-oxidation effect exerted by baicalin. In addition, ectopic expression of DEPP in HCT116 cells significantly induced the activity of senescence-associated β-galactosidase (SA-β-Gal) in tumor cells regulated by Ras/Raf/MEK/ERK signaling pathway. Knockdown of DEPP by RNA interference efficiently counteracted the baicalin-mediated growth inhibition, senescence and cell cycle arrest in cancer cells. Importantly, in a xenograft mouse model of human colon cancer, we further confirmed that baicalin treatment dramatically inhibited tumor growth, which was due to the induction of tumor cellular senescence via the upregulation of DEPP and the activation of Ras/Raf/MEK/ERK signaling in vivo. In addition to baicalin treatment, we found that the hypoxia-response protein DEPP functions as a positive regulator involving the regulations of Ras/Raf/MEK/ERK signaling pathway and inhibition of human colon cancer by other anti-oxidative drugs, such as curcumin and sulforaphane, resulting in tumor cellular senescence. These results collectively suggest that baicalin upregulates the expression of DEPP and activates its downstream Ras/Raf/MEK/ERK and p16^INK4A^/Rb pathways by acting as an antioxidant, leading to senescence in colon cancer cells.

## Introduction

A growing amount of evidence has demonstrated that senescence is a crucial tumor-suppressive approach in cancer prevention and treatment^1–5^. It has now been explicated that cancer cells can be induced to undergo senescence by multiple therapeutic treatments such as chemotherapeutic drugs, radiation, or hypoxia^6–11^. Hence, therapy-induced senescence (TIS), usually related to multiple stimuli like oxidative stress, DNA damage, telomere erosion and oncogene expression^4^, becomes a promising approach in preventing continued tumor growth^12^. Recently, evidence suggests that oncogene Ras, an upstream adaptor of the Ras/Raf/MEK/ERK pathway, is relevant for the accumulation of p16^INK4A^ and dephosphorylation of pRb, thereby promoting cellular senescence^13^. This pathway, considered to be a kind of oncogene-induced senescence, is thought to be a crucial tumor-suppressor mechanism for plus incentive such as chemopreventive agents or therapeutic drugs^14^. Combined with the above background, specific mode of action on oncogene activity is necessary for further investigation of senescence induction in cancer therapy.

Existing research showed that ROS level affects the biological processes of tumors, such as apoptosis, genomic instability and neovasculation^15^. On one hand, low ROS level endows tumor cells with properties beneficial for their growth and survival, including radioresistance, chemoresistance and immune evasion^16^. On the other hand, low ROS level has been validated as an effective target for cancer therapy^16,17^. Covering most cases, senescence is usually related to an induction of ROS. But the microenvironment of tumor cells is naturally hypoxic, which on the contrary generated the production of ROS. High level of ROS is required for the stabilization of HIF-1α, which instead activates VEGF to promote the proliferation of tumor cells^18^. The easiest way to reduce ROS is high degree of hypoxia. Nevertheless, only concepts related to oncogene, such as Ras, indirectly support that high degree of hypoxia may induce senescence in cancer cells, without clear experimental validation^19^. Furthermore, several hypoxia-response genes involved in cell cycle control, stress response and angiogenesis have been identified in the malignant glioma cell line U-251, such as *Cyclin G2*, *v-Fos*, *DNA damage-inducible transcript 3*, *Glutathione S-transferase* and *DEPP*^20^. However, none of these genes have been reported to be relevant for cellular senescence. According to our preliminary results from microarray analysis, among these hypoxia-response genes, only *DEPP* is upregulated in response to baicalin in tumor cells. Furthermore, another study suggested that the induction of DEPP increases the level of phosphorylated ERK and its target transcription factor Elk-1^21^. However, the functional role of DEPP in senescence induction in cancer cells mediated by baicalin is unclear.

Baicalin (7-glucuronic acid-5,6-dihydroxy-flavone) is a type of flavonoid extracted from *Scutellaria* root with prominent biological activities including anti-oxidation, anti-cancer, anti-inflammation with little toxicity to normal tissues^22–24^. A previous study revealed that cell cycle arrest in colon carcinoma was induced by baicalin treatment, without obvious apoptosis induction^22^, whereas the mechanism responsible for this molecular process is still disputed. Further investigation on the anti-oxidation activity and senescence induction exerted by baicalin is needed.

In the current study, we investigated the biological processes between baicalin administration and senescence induction in colon cancer cells in vitro and in xenograft models. We illustrated that decreased ROS level mediated upregulation of DEPP and DEPP expression definitely elicits cellular senescence in colon cancer cells depended on the functional activation of Ras/Raf/MEK/ERK and p16^INK4A^/Rb signaling pathways. Our results identified that induction of tumor cellular senescence is an effective and promising therapeutic strategy mediated by baicalin, involving the regulation of DEPP as well as its anti-oxidative effect.

## Results

### Baicalin-Induced Senescence in Colon Cancer Cells

**Fig. 1:**
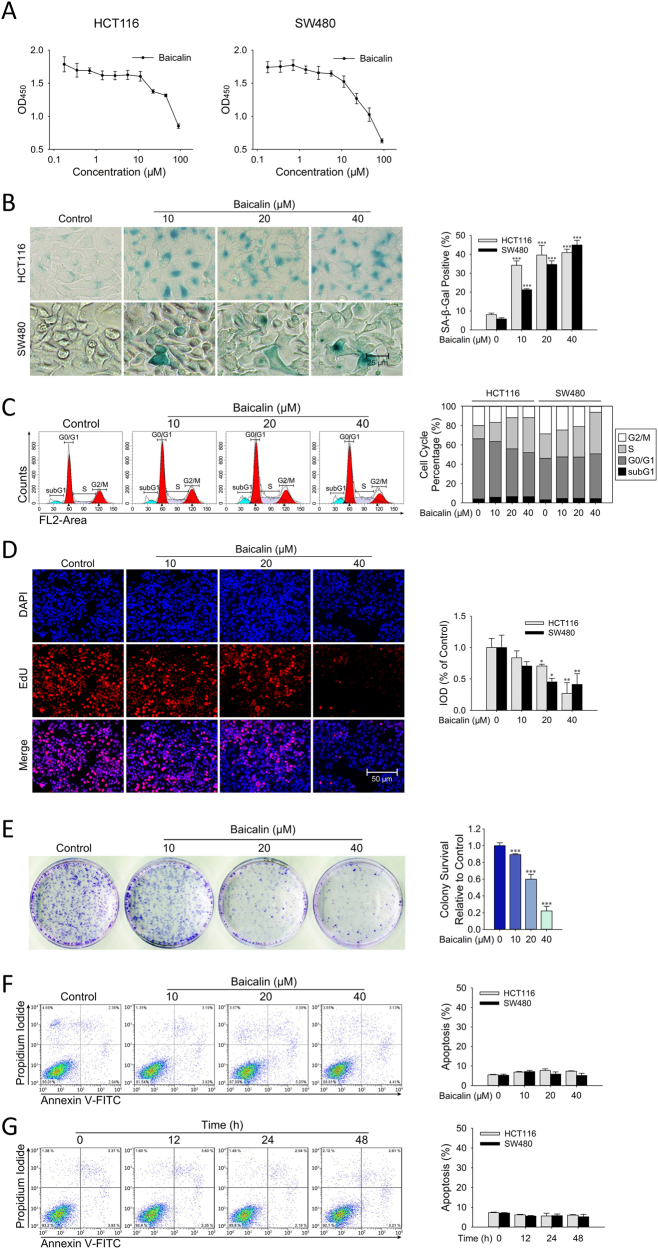
Baicalin-induced senescence in colon cancer cells. **a** HCT116 and SW480 were incubated with the indicated doses of baicalin for 24 h. The mean value of OD450 is shown as mean ± SD of six wells. Shown are representative of 5 independent repeated experiments. **b** SA-β-Gal staining was performed in HCT116 and SW480 cells treated with baicalin. Shown are representative of four experiments. The quantifications of SA-β-Gal staining are means ± SD. **c** Flow cytometric analysis of DNA content was performed in HCT116 and SW480 cells treated with baicalin. The cell cycle distribution is represented as the percentage of cells in each cycling phase (subG1, G0/G1, S, G2/M), the data are representative of three experiments. **d** EdU staining was performed in HCT116 and SW480 cells treated with baicalin. Shown are representative of three experiments. The quantifications of integrated optical density are means ± SD. **e** Colony formation assay was implemented in HCT116 cells treated with baicalin. Shown are representative of 3 experiments. The quantifications of colonies formed are means ± SD. **f**, **g** Flow cytometric analysis of Annexin V/PI double staining was performed in HCT116 cells treated with baicalin. The percentage of apoptotic cells are representative of three experiments. (*P ≤ 0.05, *P ≤ 0.01 and ***P ≤ 0.001 versus the control group)

Previous study revealed that baicalin-induced cell cycle arrest in colon carcinoma cells^22^. In CCK-8 assay, baicalin inhibited the viability of HCT116 and SW480 colon cancer cells (Fig. 1a). To further validate whether the inhibition of cancer cells mediated by baicalin is due to its induced senescence in human colon cancer cells, HCT116 and SW480 treated with baicalin at different concentrations for 48 h and then the acidic β-galactosidase activity was analyzed by senescence-associated β-galactosidase (SA-β-gal) staining. As shown in Fig. 1b, treatment with baicalin at concentrations of 10–40 μM led to significant increase of the percentages of SA-β-gal-positive cells with a dose-dependent manner. Cell cycle distribution analysis revealed that a progressive increase of cells in S-phase and quantity unchanged in subG1-phase at all doses was observed in colon cancer cells (Fig. 1c). EdU staining also verified that the proliferation of cancer cells was restricted in S-phase (Fig. 1d). In a colony formation assay, baicalin treatment significantly reduced the numbers of colonies in colon cancer cells at all doses tested (Fig. 1e). Annexin V/PI double staining was employed to determine whether baicalin has the ability to induce cell apoptosis. As shown in Fig. 1f, g, baicalin treatment did not increased apoptotic cells in human colon cancer cells. These data revealed that baicalin-induced senescence in colon cancer cells without obvious detection of apoptosis.

### Effects of Baicalin on ROS Level and SOD Activity in Colon Cancer Cells

**Fig. 2:**
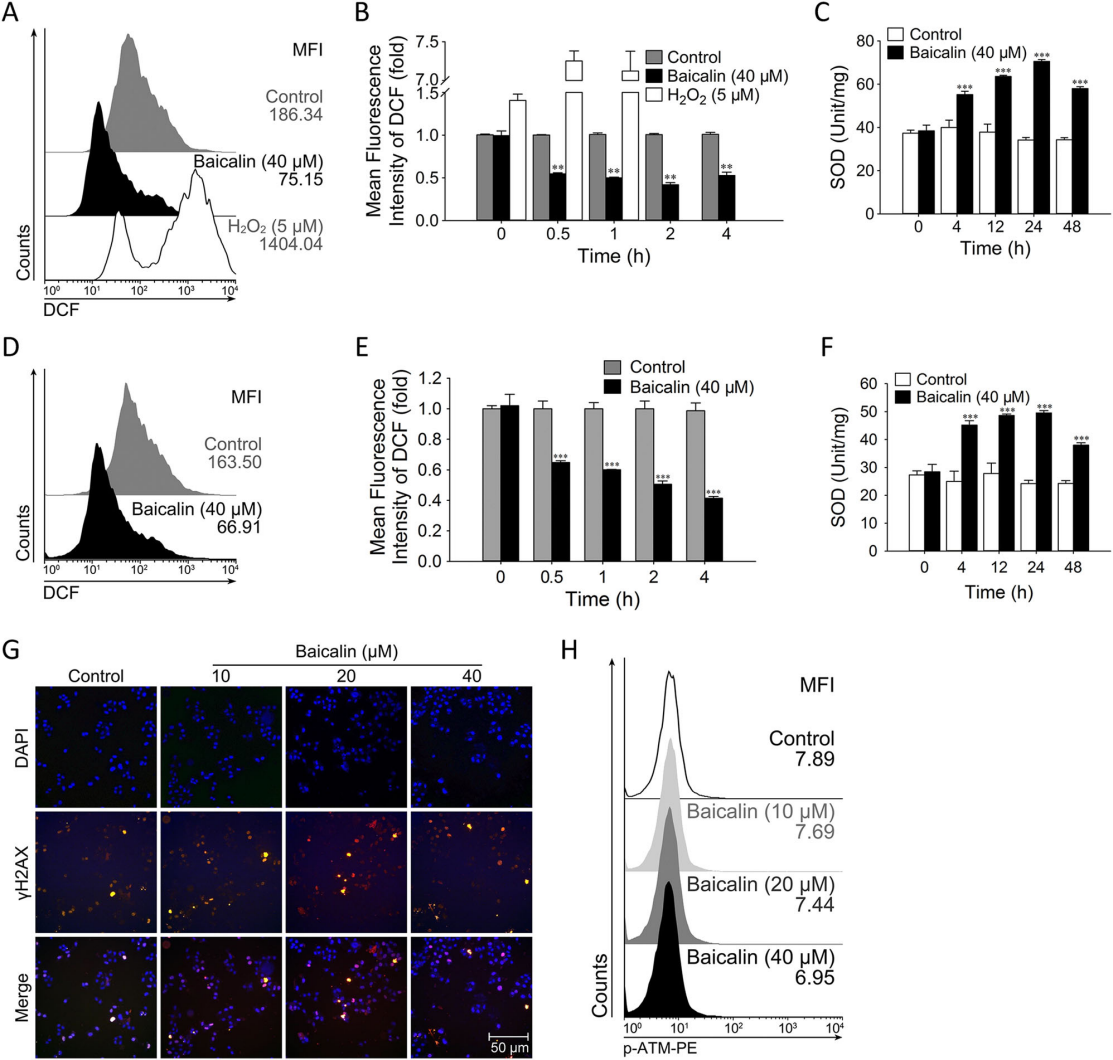
Effects of baicalin on ROS level and SOD activity in colon cancer cells. **a** Cells were incubated with the indicated doses of baicalin and H_2_O_2_ for indicated times. The represented results were obtained 4 h after treatment. The cells were stained with DCFH-DA and analyzed by flow cytometry to determine ROS level. Shown are representative of 4 experiments. **b** The mean fluoresce intensity of DCF is shown as mean values ± SD of four independent experiments. **c** The data for SOD activity in HCT116 cells treated with 40 μM baicalin were normalized to controls and are shown as the means ± SD (*n* = 4). **d**–**f** The same protocols for testing ROS level and SOD activity were conducted in SW480 cell line. **g** Double staining for the DNA damage response marker γH2AX (red) and DAPI (blue) in HCT116 cells treated with vehicle and indicated concentrations of baicalin. Shown are representative of four experiments. **h** Cells treated with vehicle or baicalin were analyzed by flow cytometry to determine the levels of p-ATM. Shown are graphical mean fluoresce intensity of 4. (**P ≤ 0.01, ***P ≤ 0.001 versus the control group)

Oxidative stress and DNA damage are frequent inducers for cellular senescence^25^. To determine which factor plays a role in baicalin-induced senescence in colon cancer cells, we tested the level of reactive oxidative species (ROS) and the activity of superoxide dismutase (SOD), as well as the protein levels of γH2AX and p-ATM in HCT116 tumor cells treated with baicalin. As shown in Fig. 2a, b, the mean fluorescence intensity (MFI) of ROS in HCT116 tumor cells treated with baicalin for 4 h was dramatically decreased compared with that of control (59.7% decrease). Meanwhile, after 24 h of baicalin treatment, the enzymatic activity of SOD was significantly increased (from 34.17 to 70.54 unit/mg; Fig. 2c). Similar results of ROS levels and SOD activity were also observed in SW480 colon cancer cells after treatment with baicalin (Fig. 2d–f). However, we did not detect a substantial increase in the phosphorylation levels of DNA damage response-associated molecule ATM and its downstream target γH2AX between control and baicalin administered cells (Fig. 2g, h). Taken together, these results clearly indicated that baicalin enhanced the activity of SOD and decreased ROS levels in colon cancer cells.

### DEPP was Upregulated by Baicalin in Cancer Cells In Vitro

**Fig. 3:**
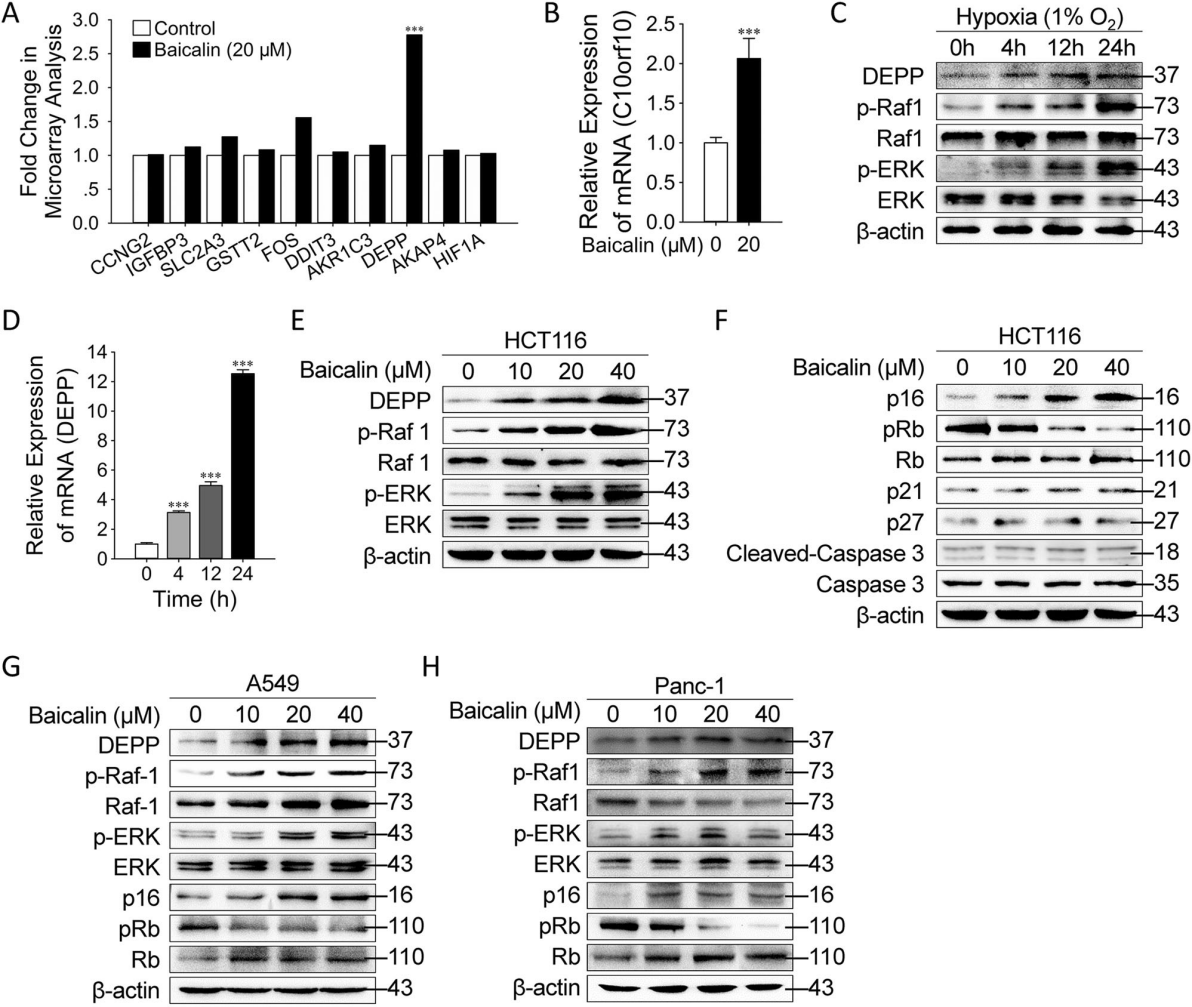
DEPP was upregulated by baicalin in cancer cells in vitro. **a** HCT116 cells treated with vehicle or baicalin were subjected to whole-genome microarray analysis. The histogram depicts a compilation of hypoxia-response genes. **b** The expression of DEPP mRNA in the vehicle-treated or baicalin-treated cells was verified by qRT–PCR. The data shown are means ± SD from three independent experiments. **c** HCT116 cells were exposed to hypoxic condition for indicated times, and the protein levels of DEPP, p-Raf1, Raf1, p-ERK, ERK, p16^INK4A^, pRb, Rb and β-actin were analyzed by western blotting. **d** The expression of DEPP mRNA in HCT116 cells exposed to hypoxic condition was verified by qRT–PCR. The data shown are means ± SD from three independent experiments. **e** HCT116 cells were treated with vehicle or baicalin at indicated concentrations, and the protein levels of DEPP, p-Raf1, Raf1, p-ERK, ERK and β-actin were analyzed by Western blotting. **f** HCT116 cells were treated with vehicle or varying concentrations of baicalin (10, 20 and 40 μM) for 24 h, and the protein levels of p16^INK4A^, pRb, Rb, p21, p27, cleaved-caspase 3 and caspase 3 were analyzed by western blotting. **g**, **h** A549 and Panc-1 cells were treated with vehicle or baicalin at indicated concentrations, and the protein levels of DEPP, p-Raf1, Raf1, p-ERK, ERK, p16^INK4A^, pRb, Rb and β-actin were analyzed by Western blotting. (***P ≤ 0.001 versus the control group)

To explore the molecular basis of tumor inhibition mediated by baicalin, we employed a genome-wide transcriptional microarray analysis of the transcriptional profile of baicalin-treated HCT116 cells. To extend our study on baicalin-induced senescence through low ROS level, we focused on the differentially expressed genes that were hypoxia-response and involved in cell cycle regulation, stress response and angiogenesis (Fig. 3a)^20^. Among those genes, only one molecule named DEPP encoded by chromosome 10 open reading frame 10 (C10orf10) that had been previously reported to be able to increase the phosphorylation of MAPK/ERK signaling, was significantly upregulated (2.78-folds). This result was further verified by quantitative reverse transcription–PCR (qRT–PCR) to determine the mRNA level of DEPP in HCT116 cells treated with baicalin (Fig. 3b). To further explore the regulation of DEPP in a hypoxic condition of tumor cells, HCT116 cells were placed into a hypoxic incubator maintained 1% O_2_. Western blotting and qRT–PCR analyses showed that the expression of DEPP was significantly upregulated (Fig. 3c,d). In addition to the increased expression of DEPP, the phosphorylation and the activation of Raf1 and ERK were dramatically induced in the colon cancer cells in response to the hypoxic condition (Fig. 3c). Furthermore, we found that the expression and activation of those molecules followed the trends as shown in the hypoxic condition in HCT116 tumor cells after the treatment of baicalin with a dose-dependent manner (Fig. 3e). Evidence demonstrates that the majority of cell cycle regulators, such as caspase 3, p21, p27, p16^INK4A^, and Rb are critically involved in the induction of cellular senescence^6,26^. However, we found that the administration of baicalin in HCT116 cells only significantly affected the protein levels of p16^INK4A^ and Rb during the induction of cellular senescence (Fig. 3f). These results suggest that the low ROS level generated by baicalin in tumor cells, resulted in the upregulation of DEPP and the activation of Ras/Raf/MEK/ERK and p16^INK4A^/Rb pathways. Similar results of DEPP upregulation and the activation of Ras/Raf/MEK/ERK and p16^INK4A^/Rb pathways were also observed in A549 (Fig. 3g) and Panc-1 (Fig. 3h) cell lines.

### Baicalin Upregulated DEPP Expression and Induced Senescence in Colon Cancer Cells in vivo

**Fig. 4:**
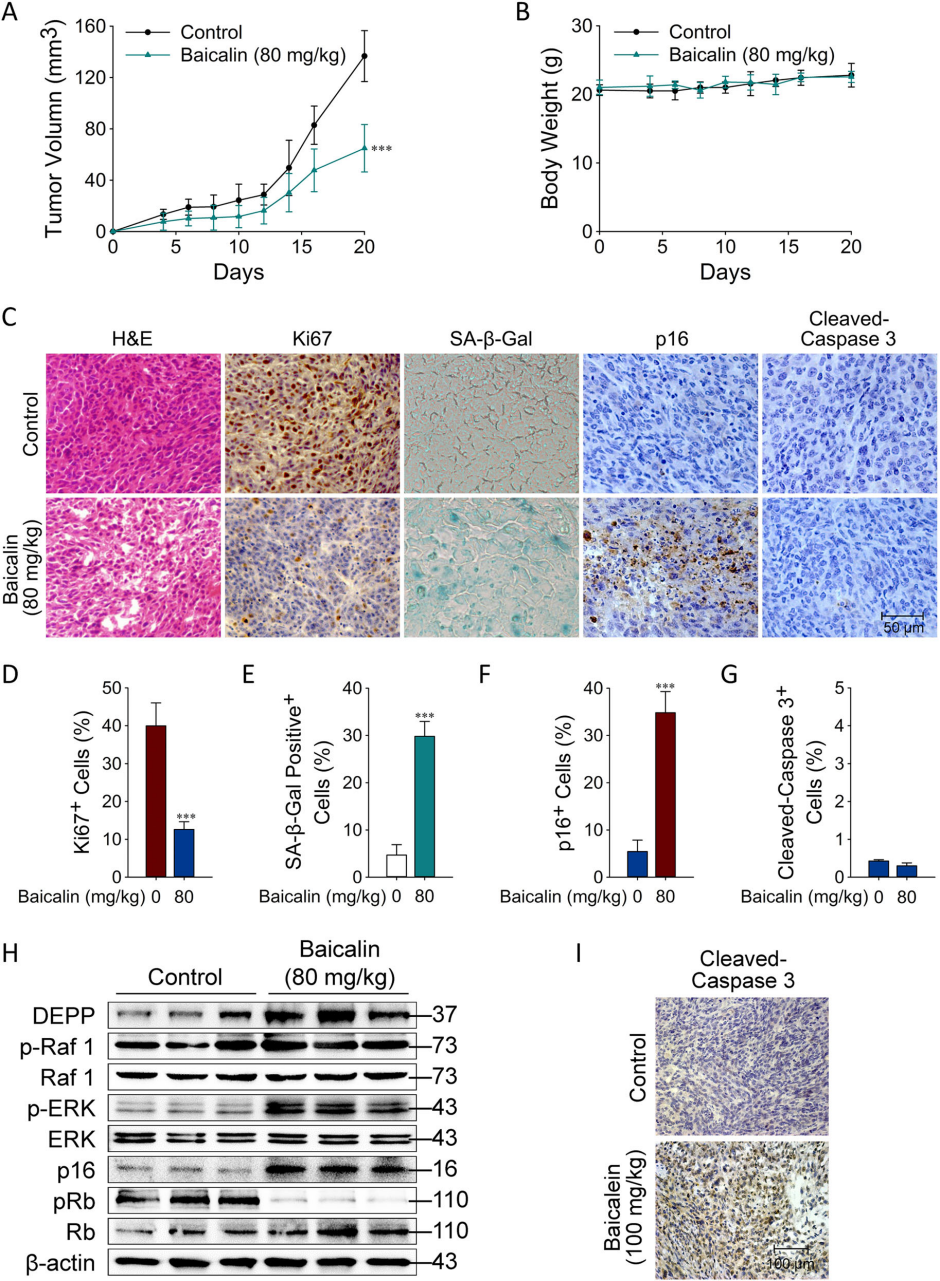
Baicalin upregulated DEPP expression and induced senescence in colon cancer cells in vivo. **a**, **b** Effects of baicalin on tumor volume and body weight. The data are representative of results obtained from 5 mice. **c** Tumor sections were subjected to hematoxylin and eosin staining, SA-β-Gal staining and IHC for Ki67, p16^INK4A^ and cleaved-caspase 3. Original magnification was 400×. Every single shown picture is representative of five sections from every group. Different sections represent different tumors. The representative images and the calculation of the percentage of positive cells are shown in **d**–**g**; the data are the means ± SD (***P ≤ 0.001). **h** Tumors were collected for western blot analysis of DEPP, p-Raf1, Raf1, p-ERK, ERK, p16^INK4A^, pRb, Rb and β-actin. Every single lane represents one tumor from an individual mouse. **i** Tumor sections were subjected to cleaved-caspase 3. Original magnification was 400×

We next performed in vivo studies in a xenograft mouse model of human colon cancer HCT116 cells to validate our in vitro findings and results. One week after the inoculation of cancer cells, baicalin was given by i.p. injection for a total of 14 days, and then the tumors were collected for evaluation of cell proliferation and signaling activation, as well as senescence induction at the end of the experiments. We observed that HCT116 tumor cells grew progressively in mice. However, treatment with baicalin significantly inhibited tumor growth without affecting body weight obviously in mice (Fig. 4a, b). Furthermore, treatment with baicalin also dramatically decreased the Ki67^+^ cell populations in tumor tissues, suggesting that baicalin inhibited tumor proliferation (Fig. 4c, d and Supplementary Fig. 1). In contrast, treatment with baicalin significantly increased the SA-β-gal-positive cell population and p16^INK4A^ expression in tumor tissues, suggesting that baicalin-induced cellular senescence in tumor cells (Fig. 4c, e, f and Supplementary Fig. 1). We further confirmed that baicalin treatment did not induce cell apoptosis (no significant changes in cleaved-caspase 3 expression were detected) (Fig. 4c, g and Supplementary Fig. 1). The antibody of cleaved-caspase 3 was confirmed to be functionally worked with baicalein which was previously proved to induced apoptosis (Fig. 4i)^27^. Consistent with the in vitro studies, the positive stain of the proliferation and senescence markers and protein level of DEPP were increased accompanying with Raf1 and ERK activation in tumor tissues in the baicalin treatment group (Fig. 4h). In addition, p16^INK4A^ was increased and the phosphorylation level of pRb was decreased after the treatment of baicalin (Fig. 4h). These data suggested that baicalin can inhibit tumor growth in vivo, which is due to the induction of tumor cellular senescence involving the upregulation of DEPP and the activation of Ras/Raf/MEK/ERK and p16^INK4A^/Rb signaling pathways.

### Knockdown of DEPP Attenuated Baicalin-Induced Senescence in Colon Cancer Cells

**Fig. 5:**
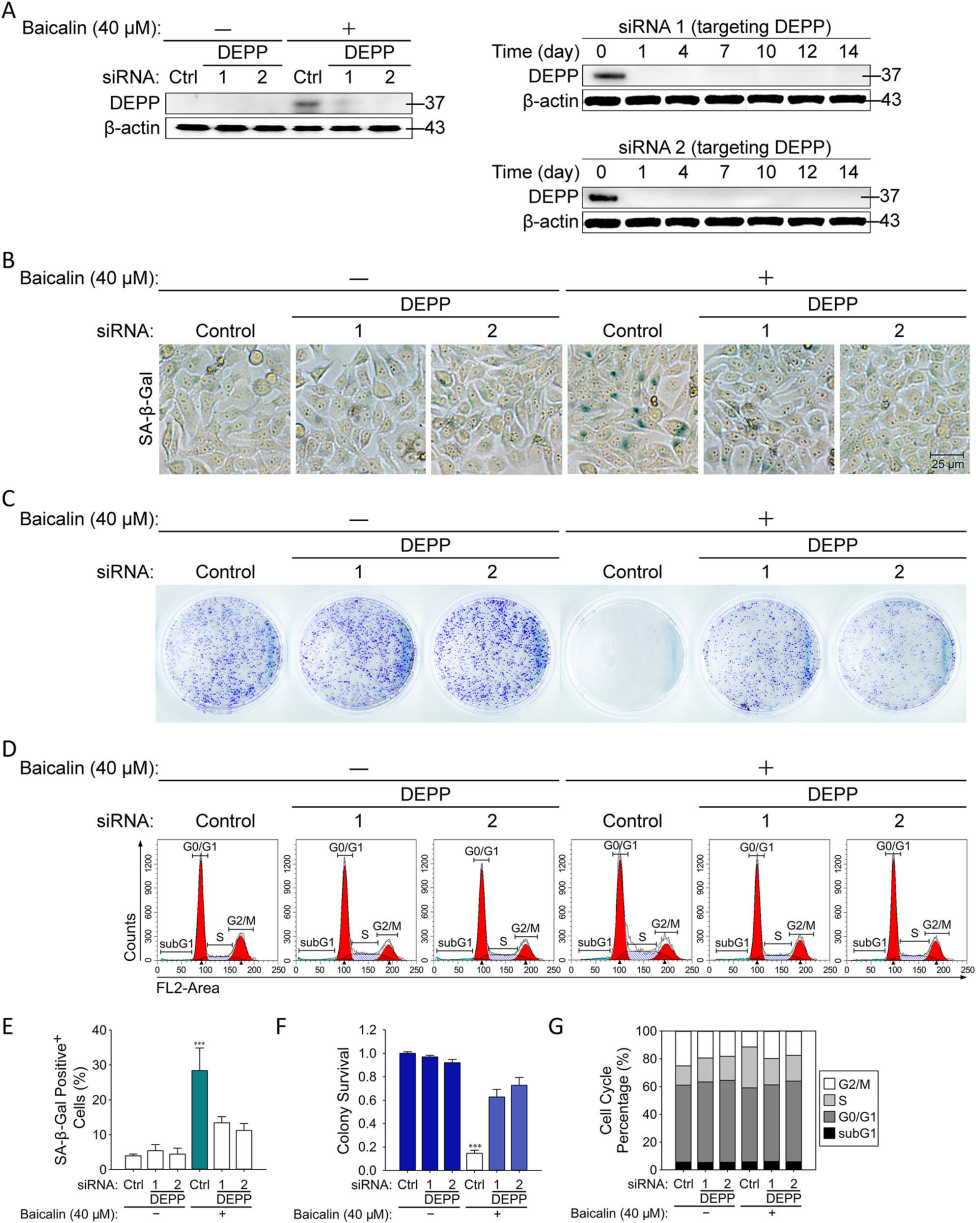
Knockdown of DEPP attenuated baicalin-induced senescence in colon cancer cells. **a** The validation of two different siRNA targeting DEPP was analyzed by western blot of DEPP and β-actin. **b** SA-β-Gal staining was performed in HCT116 cells transfected with siDEPP during baicalin-induced senescence. Shown are representative of four experiments. The quantifications of SA-β-Gal staining are shown in **e**; the data are means ± SD. **c** Flow cytometric analysis of DNA content was performed in HCT116 cells transfected with siDEPP during baicalin-induced cell cycle arrest. Data are representative of three experiments. The cell cycle distribution is represented as the percentage of cells in each cycling phase (G0/G1, S, G2/M) in **f**. **d** Colony formation assay was implemented in HCT116 cells transfected with siDEPP during baicalin-induced proliferation inhibition. Shown are representative of three experiments. The quantifications of colonies formed are shown in **g**; the data are means ± SD. (***P ≤ 0.001 versus the control group)

To understand the functional role of DEPP in regulation of tumor cellular senescence mediated by baicalin, HCT116 cells were transfected with siRNA targeting DEPP and then cultured with baicalin to determine senescence induction and tumor growth. We firstly verified that siRNA targeting DEPP stably depleted the expression of DEPP (Fig. 5a). Treatment with only baicalin led to observably increased numbers of SA-β-gal-positive cells in HCT116 tumor cells (Fig. 5b, e). In the colony formation assay, baicalin was found to significantly reduce the number of colonies formed by HCT116 cells (Fig. 5c, f). Cell cycle distribution analysis revealed that a progressive increase of cells in S-phase and a decrease in G2/M-phase was observed in colon cancer cells (Fig. 1d, g). Furthermore, by contrast, knockdown of DEPP expression with two different targeting siRNA significantly prevented senescence induction (SA-β-Gal^+^ cells changed from 28.3 to 13.4 and 11.2%, respectively), decreased the S-phase cell cycle arrest and increased the numbers of cells in G0/G1 phase, as well as increased the number of colonies formed by HCT116 colon cancer cells (Fig. 5b–g). These data further suggested that reduced expression of DEPP partially converted baicalin-induced senescent cancer cells to regain their proliferation ability. Our results collectively indicate that DEPP is a key regulator in the process of baicalin-induced senescence in tumor cells.

### DEPP Controlled Baicalin-Induced Senescence and the Activation of Ras/Raf/MEK/ERK and p16^INK4A^/Rb Signaling Pathways in Cancer Cells

**Fig. 6:**
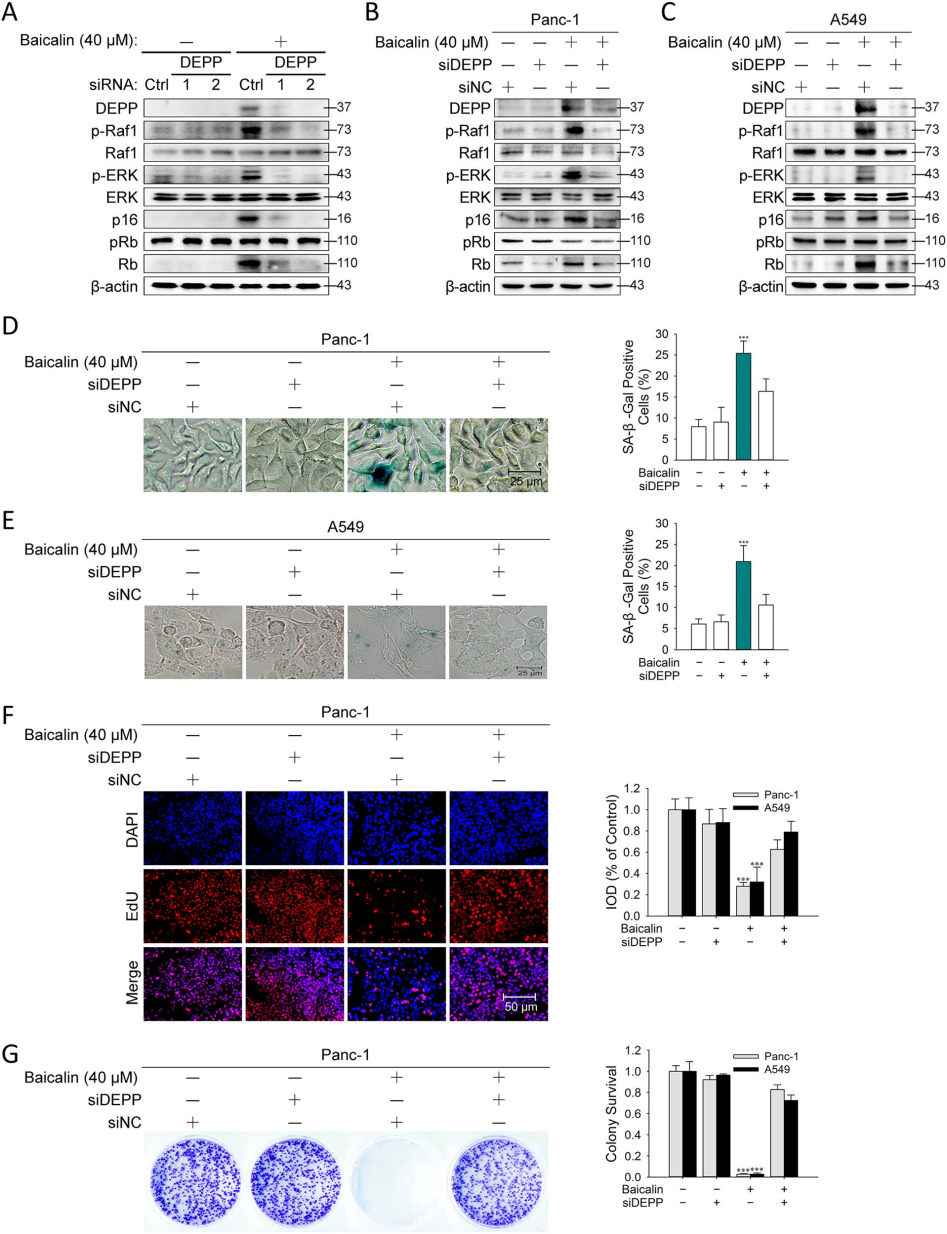
DEPP controlled baicalin-induced senescence and the activation of Ras/Raf/MEK/ERK and p16^INK4A^/Rb signaling pathways in cancer cells. HCT116 cells transfected with either control siRNA or siDEPP (no. 1 and 2) were exposed to baicalin (40 μM), and 24 h later, immunoblotting analysis **a** was performed. Panc-1 and A549 cells transfected with either control siRNA or siDEPP (no. 2) were exposed to baicalin (40 µM), and 24 h later, immunoblotting analysis **b**, **c** were performed. **d**, **e** SA-β-Gal staining was performed in Panc-1 and A549 cells treated with baicalin. Shown are representative of three experiments. The quantifications of SA-β-Gal staining are means ± SD. **f** EdU staining was performed in Panc-1 and A549 cells treated with baicalin. Shown are representative of three experiments. The quantifications of integrated optical density are means ± SD. **g** Colony formation assay was implemented in Panc-1 and A549 cells treated with baicalin. Shown are representative of 3 experiments. The quantifications of colonies formed are means ± SD. (***P ≤ 0.001 versus the control group)

To investigate the causative role of DEPP in the regulation of baicalin-induced senescence, we evaluated whether the inhibition of DEPP expression by RNA interference affects Ras/Raf/MEK/ERK signaling, p16^INK4A^/Rb pathway and senescence induction in human cancer cells. When DEPP expression in tumor cells was knocked down, we observed that Ras/Raf/MEK/ERK and p16^INK4A^/Rb pathways were apparently inactivated in HCT116, Panc-1 and A549 cell lines (Fig. 6a–c). The phenomena that senescence induced by baicalin dependently of DEPP upregulation were also observed in Panc-1 and A549 cell lines with assessment of SA-β-Gal activity (Fig. 6d, e), EdU staining (Fig. 6f) and colony formation assay (Fig. 6g). These results indicated that DEPP participates in the activation of Ras/Raf/MEK/ERK and p16^INK4A^/Rb signaling pathways and senescence induction mediated by baicalin tumor cells.

### Ras/Raf/MEK/ERK Signaling Pathway was Involved in DEPP-Mediated Tumor Cellular Senescence in Colon Cancer Cells

**Fig. 7:**
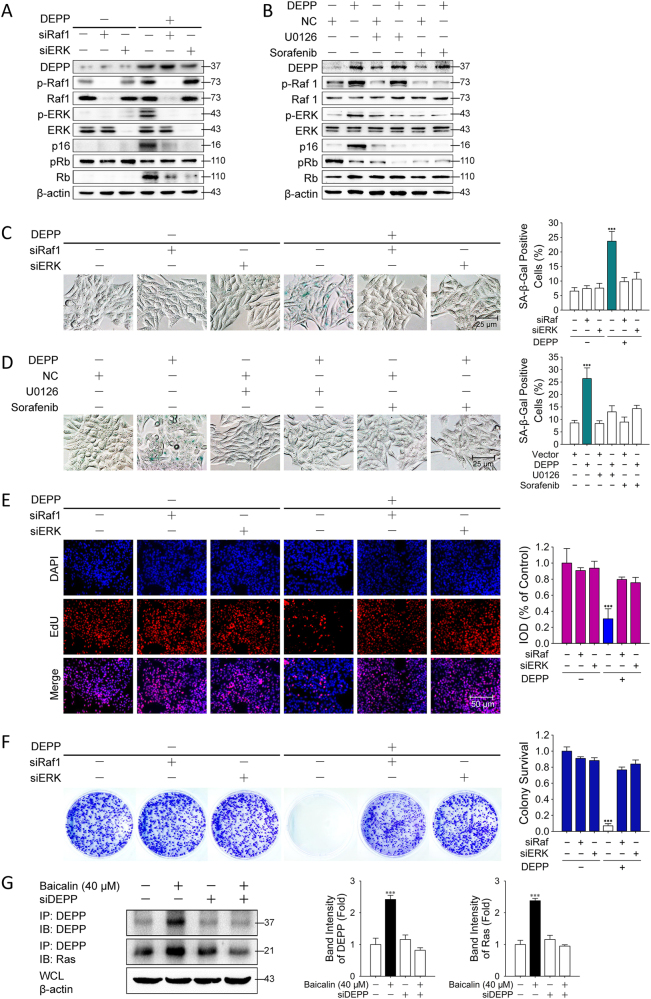
Ras/Raf/MEK/ERK signaling pathway was involved in DEPP-mediated tumor cellular senescence in colon cancer cells. HCT116 cells transfected with pcDNA3.1 either blank or loaded the cDNA of DEPP were treated with siRNA targeting Raf1 and ERK, then immunoblotting analysis **a** and SA-β-Gal staining **c** were carried out. Shown are representative of 4 experiments. HCT116 cells transfected with pcDNA3.1 either blank or loaded the cDNA of DEPP were treated with Sorafenib or U0126 for 24 h, then immunoblotting analysis **b**, SA-β-Gal staining **d**, EdU staining **e** and colony formation assay **f** were carried out. Shown are representative of 4 experiments. The quantification of SA-β-Gal and crystal violet staining represents the means ± SD. The integrated optical density of EdU staining represents the means ± SD. (***P ≤ 0.001 versus the control group). **g** Immunoblotting of Flag-DEPP immunoprecipitates from HCT116 cells with or without the treatment with baicalin or siDEPP. The binding of DEPP to Ras in cells is presented relative to that in the untreated cells. Shown are representative of three experiments

To understand the interaction between DEPP and Ras/Raf/MEK/ERK signaling pathway, siRNAs targeting Raf1 and ERK were used to inhibit the expression of Raf1 and ERK. Overexpression of DEPP distinctly increased the protein levels of p-Raf1, p-ERK and p16^INK4A^, and decreased the expression of pRb in tumor cells (Fig. 7a, b). After knockdown of Raf1 or ERK, baicalin failed to influence the p16/Rb pathways (Fig. 7a). U0126 is a highly selective inhibitor for MEK1/2 and sorafenib is a novel multikinase inhibitor that targets the Raf family. The effects of DEPP on Ras/Raf/MEK/ERK and p16^INK4A^/Rb pathways were validated by the loss-of-function strategy with respective U0126 and sorafenib treatment in the presence or absence of DEPP overexpression. DEPP overexpression did not change the contents of p16, Rb after the treatments with those two inhibitors (Fig. 7b). We then investigated whether the ectopic expression of DEPP and inhibition of Ras/Raf/MEK/ERK signaling affect tumor cellular senescence. Overexpression of DEPP distinctly increased the percentage of SA-β-gal-positive cells (from 8.4 to 25.7%) (Fig. 7c, d). After knockdown of Raf1 or ERK, the numbers of senescent HCT116 cells were dramatically decreased (from 23.7 to 9.8 and 10.7%, respectively; Fig. 7c). Same results obtained after the treatments with U0126 and sorafenib (Fig. 7d). Overexpression of DEPP also obviously increased the fluorescence intensity of cells stained with EdU and decreased the number of colonies of HCT116 cells (Fig. 7e, f). The knockdown of Raf1 and ERK prevented the reduction EdU with recovered colony numbers (Fig. 7e, f). Taken together, these results indicate that DEPP-induced senescence was dependent on the activation and regulation of Ras/Raf/MEK/ERK signaling pathway. KRas acts as a molecular on/off switcher utilizing protein dynamics. Once it is allosterically activated, it recruits and activates other cell signaling receptors such as Raf1^28,29^. We identified the physical interaction between DEPP and Ras with the co-immunoprecipitation assay. We found that the precipitated proteins co-immunoprecipitated with anti-DEPP showed specific enrichment of Ras protein (Fig. 7g). These results indicated that DEPP combined KRas to control Ras/Raf/MEK/ERK signaling pathway which is necessary in DEPP-mediated p16/Rb activation.

### Curcumin and Sulforaphane Upregulated DEPP Expression and Induced Senescence in HCT116 Cells

**Fig. 8:**
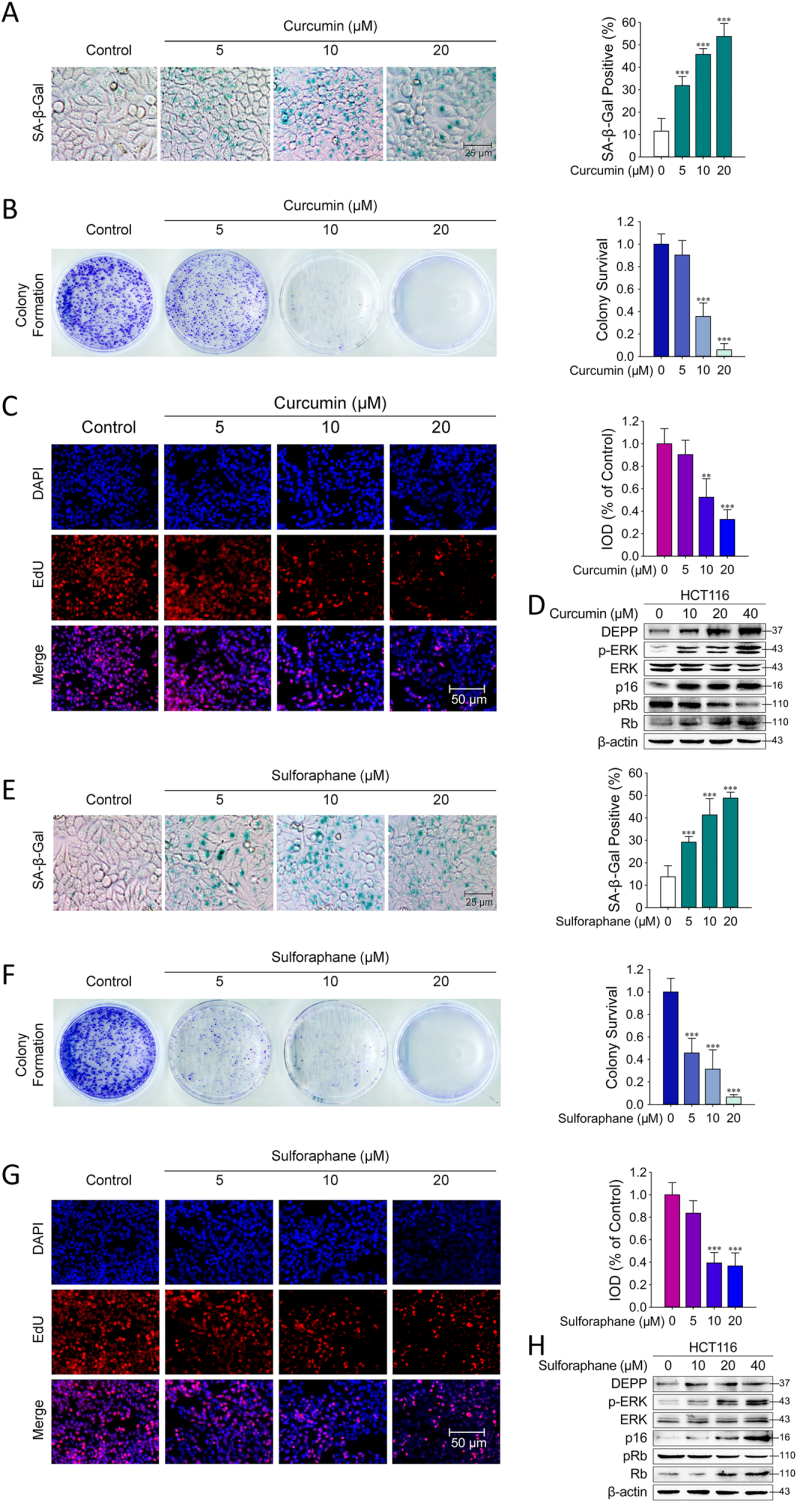
Curcumin and sulforaphane upregulated DEPP expression and induced senescence in HCT116 cells. SA-β-Gal staining, colony formation assay and EdU staining was performed in HCT116 cells treated with curcumin or sulforaphane in. The images are representative of 4 experiments. The representative images and quantifications of SA-β-Gal staining are shown in **a**, **e**. The cell cycle distribution is represented as the percentage of cells in each cycling phase (G0/G1, S, G2/M) in **b**, **f**. The fluorescence intensity of EdU is represents as the images of immunofluorescence staining and the integrated optical density. The data are the means ± SD (**P ≤ 0.01, ***P ≤ 0.001). Cells treated with curcumin or sulforaphane were collected for western blot analysis of DEPP and associated signaling proteins. **d** Curcumin. **h** Sulforaphane

To verify whether other natural anti-oxidative drugs also exert a similar effect on colon cancer cells similar to baicalin, we treated HCT116 cells with curcumin or sulforaphane at concentrations of 5–20 μM for 48 h and then acidic β-galactosidase activity was analyzed by SA-β-gal staining. As shown in Fig. 8a, b, treatment with curcumin or sulforaphane led to significantly increased SA-β-gal^+^ HCT116 cells. Results of colony formation assay and EdU staining were also verified that curcumin-induced or sulforaphane-induced senescence in HCT116 cells (Fig. 8a–c, e–g). In addition, Western blotting analyses showed that treatment with curcumin or sulforaphane resulted in sharp increases of DEPP, p-ERK, p16^INK4A^ protein levels with the significant decrease of phosphorylation level of pRb (Fig. 8d, h). These results revealed that both curcumin and sulforaphane had the ability to induce senescence with DEPP-dependent manner.

## Discussion

High degree of hypoxia can induce cell cycle arrest in a variety of cell types^11,19^. However, whether hypoxia functions as a promoter of tumorigenesis or a target for cancer therapy remains controversial^16,17,30^. Our current study demonstrated that baicalin intensively induced hypoxia-like phenomenon of low ROS level in colon cancer cells, due to the enhanced SOD activity. While definitely senescent phenotypes were also observed after the administration of baicalin. Various types of evidences proved that baicalin induced a permanent S-phase cell cycle arrest in human colon cancer cells. Therefore, we hypothesized that genes showing increased expression in response to hypoxia possibly play an important role in the regulation of baicalin-induced low ROS level and senescence in colon cancer cells. Covering the most common cases, hypoxia induces a G1/S arrest, which is dependent on HIF mediated expression of cell cycle regulators p21 and p27^10,11,31^. Besides the HIFs, several other genes including DEPP, which acts on cell cycle or stress response, can also influence cancer cells. Also the natural property of hypoxia in cancer cells determined that cancer cells will benefit from the ROS production^18^. In the current study, we found that DEPP was dramatically upregulated in multiple kinds of cancer cells in response to baicalin treatment. Furthermore, knockdown of DEPP by RNA interference resulted in an attenuation of baicalin-induced cellular senescence, cell cycle arrest and growth inhibition in tumor cells. Likewise, the ectopic expression of DEPP intensively induced tumor cellular senescence. These studies strongly suggest that baicalin-induced tumor inhibition is through the regulation of DEPP.

Compelling evidence demonstrates that hyperactivation of Ras/Raf/MEK/ERK functions as a downstream pathway of carcinostatic signals, comprising p16^INK4A^/Rb, p21, p27 and p53 to inhibit tumor development^32–35^. Recent studies suggest that accumulation of DEPP leads to the activation of the transcription factor ELK1 through phosphorylation of MAPK/ERK pathway^21^. Therefore, we hypothesized that the increase of DEPP production mediates the link between the low ROS level and senescence, especially senescence in response to the activation of Ras/Raf/MEK/ERK pathway. According to our microarray analysis and functional verification studies, the bridge between baicalin treatment and Ras/Raf/MEK/ERK signaling were turned out to be DEPP. Of note, baicalin treatment led to an increase in the phosphorylation of Ras binding protein Raf1 and its downstream protein ERK. At the same time, baicalin selectively influenced p16^INK4A^/Rb pathway rather than p53, p21, p27 during the induction of tumor cellular senescence. Meanwhile, the inhibition of DEPP led to a significant reduction in the activation of Ras/Raf/MEK/ERK signaling and p16^INK4A^ expression. Simultaneously, the overexpression of DEPP induced a dramatic increase of senescent cell population, while the inhibition of Raf and ERK by inhibitors can decrease p16^INK4A^ expression and percentage of senescent cells induced by DEPP expression. Co-immunoprecipitation assay revealed that DEPP physically interacted with Ras after baicalin treatment. Taken together, these results strongly indicate that DEPP-mediated activation of Ras/Raf/MEK/ERK and p16^INK4A^/Rb pathways plays a critical role in the senescence induction in cancer cells.

Our results showed that baicalin treatment strongly increases the activity of SOD and decreases the level of ROS. The low ROS level induced by baicalin is associated with the expression of DEPP and the activation of Ras/Raf/MEK/ERK signaling pathway. As a matter of fact, many monomers possess the ability of anti-oxidation by potentiating the activity of superoxide dismutase. To verify the link between antioxidant molecules and the upregulation of DEPP, we then tested two other monomers, curcumin and sulforaphane, widely applied in traditional Chinese medicine^36,37^. The SA-β-Gal-positive ratio, DEPP production, phosphorylation of ERK and accumulation of p16^INK4A^ were all ascended up in colon cancer cells after the administration of curcumin or sulforaphane, similar to the results obtained from baicalin treatment. From a therapeutic point of view, anti-oxidative drugs modulating ROS level not only allow reinstatement of the tumor-suppressive function of senescence, but also benefit classical treatment approaches. Our studies also suggest that anti-oxidative drugs show a promising potential to exert a tumor-suppressive action, and provide optimism for eventually colon cancer cure.

In summary, our investigations demonstrated that the increased expression of DEPP in colon cancer cells acts as a key regulator for senescence induction mediated by baicalin. Moreover, inhibition of DEPP attenuates baicalin-induced tumor cellular senescence via down-regulation of Ras/Raf/MEK/ERK and p16^INK4A^/Rb signaling. These findings have established DEPP as a key node to regulate senescence induction, which could be a novel target for cancer treatment. However, several missing links remain to be solved in our future studies including the molecular mechanism responsible for baicalin-mediated upregulation of DEPP.

## Materials and Methods

### Chemicals and Antibodies

Baicalin was obtained from Jiangsu Institute for Food and Drug Control, with a typical purity of more than 93%. Crystal violet was purchased from Nanjing Chemical Reagent Co., LTD (Nanjing, China). Antibodies specific for pRb (8516), Rb (9313), p21 (2947), p27 (3686), Ras (3339), Raf1 (53745), p-ERK (9101) and ERK (4695) were purchased from Cell Signaling Technology (Danvers, MA, USA). Antibodies for DEPP (25833-1-AP) were purchased from Proteintech (Rosemont, IL, USA). The antibody for p16^INK4A^ (41296), cleaved-caspase 3 (40500), caspase 3 (42437), β-actin (4970), and p- Raf1 (11006), goat anti-mouse IgG secondary antibody (3012) and goat anti-rabbit IgG secondary antibody (3032) were purchased from SAB Signalway Antibody (Nanjing, China). The antibody for Ki67 (AB9260) was purchased from Merck Millipore (Billerica, MA, USA). The SP Immunohistochemical secondary antibody kit-Rabbit was purchased from MaiBio (Nanjing, China). The anti-γH2AX (S139)-PE antibody was purchased from eBioscience (Hatfield, UK).

### Cell Lines and Culture

HCT116, SW480, A549 and Panc-1 cell lines were obtained from the American Type Culture Collection (Rockville, MD, USA). The cells were grown in RPMI 1640 medium (Gibco, Grand Island, NY, USA) supplemented with 10% fetal bovine serum with antibiotics (100 U/ml penicillin and 0.1 mg/ml streptomycin).

### CCK-8 Assay

Cells were seeded into 96-well plates at density of 5 × 10^3^ cells per well, incubated overnight and treated with various concentrations of baicalin for 24 h. Thereafter, 10 μl of CCK-8 solution was added to each well, then incubated the plates at 37 °C for 2 h. The optical density (OD) was recorded at 450 nm.

### EdU Staining

Proliferation of cells was cytochemically detected according to the manufacturer’s instructions (C10310-3, Ribobio, Guangzhou, China). In brief, the tumor cells were incubated with the EdU staining buffer, fixed in 4% polyformaldehyde and stained the nuclear with DAPI. The stained cells were observed and photographed under microscope. In addition, integrated optical density (IOD) was used to assess EdU-positive cells with ImageJ software (Bethesda, MD, USA).

### FACS Analysis

Cells were fixed in 70% ethanol at 4 °C overnight and then stained with Cell Cycle Assay Kit (Vazyme, Nanjing, China) for 10 min. Furthermore, cells were collected after the treatment of baicalin and stained with Annexin V-FITC/PI Apoptosis Detection Kit (Vazyme) or incubated with anti-phospho-ATM-PE (Ser1981) antibody (Merck Millipore). All the cells were analyzed by flow cytometry (BD FACSCalibur, Franklin Lakes, NJ, USA).

### Microarray Analysis

Extraction of total RNA was performed with TRIzol according to the manufacturer’s instructions. The RNA samples were further purified with RNeasy Mini Kit (Qiagen, Dusseldorf, Germany). Agilent whole-genome oligo microarray gene expression analysis was performed in HCT116 cells for a total of six times. The arrays were as follows: Array 1–3: untreated cells; Array 4–6: baicalin-treated cells. For each sample, 200 ng total RNA was reverse transcribed, linear amplified, and labeled with Cy3 using Quick Amp Labeling Kit, One-Color (Agilent Technologies, Santa Clara, CA, USA), according to manufacturer’s instructions. After labeling, samples were measured on a Nanodrop ND-1000 (Thermo Scientific, Waltham, MA, USA) microarray module for labeling efficiency and quantification. The samples were then hybridized on Agilent 4 × 44 K whole-human genome GE arrays (Agilent Technologies) at 65 °C for 17 h. After washing in GE washing buffer, each slide was scanned with Agilent Microarray Scanner G2565BA. Feature extraction software was used to convert the image into gene expression data. Data were normalized by the Linear Lowess method. Genes that were 1.5-fold differentially expressed on three arrays were scored as significant. Furthermore, only genes with *P*-values ≤0.05 based on Student’s *t*-test were selected. Mean fold change is the mean of three arrays.

### Reverse Transcription–PCR

Total RNA was extracted at 24 h after baicalin treatment using an RNeasy Mini kit (74104, Qiagen) and was used for reverse transcription according to the manufacturer’s instructions. The sequences of the primers used for PCR are as follows: GAPDH, 5′-GGGAAACTGTGGCGTGAT-3′ (forward) and 5′-GAGTGGGTGTCGCTGTTGA-3′ (reverse) and DEPP, 5′-ATACGTCCTGTGGTGGCATTG-3′ (forward) and 5′-CCTGATTCCCGTTCCCTGAT-3′ (reverse). The quantification of real-time PCR products was performed using QuantiFast Probe RT–PCR Kit (204443, Qiagen). The housekeeping gene GAPDH was used as the internal control for RNA integrity and expression normalization.

### Western blotting

Cell lysates were prepared and transferred to a polyvinylidene fluoride membrane (IPVH00010, Merck Millipore). The blot was then probed with primary antibodies followed by incubation with appropriate horseradish peroxidase-conjugated secondary antibodies. The signal was visualized by the Immobilon Western Chemiluminescent HRP Substrate (Merck Millipore). All graph of Western blotting shown are representative of three experiments.

### Colony formation assay

Cells were seeded into 6 cm diameter plates (400 cells per plate) and cultured for 48 h before treatment. After the treatment with gradient concentrations of baicalin, cells were then cultured for 2 weeks. The cell colonies on the plate were fixed with 4% paraformaldehyde, stained with crystal violet and then counted.

### Assessment of Senescence-Associated β-Galactosidase (SA-β-gal) Activity

SA-β-gal activity at pH 6.0 was cytochemically detected according to the manufacturer’s instructions (C0602, Beyotime, Nanjing, China). In brief, the tumor cells were fixed with the fixative solution and stained with the staining solution provided with the kit. The stained cells were observed and photographed under microscope. Total cell numbers and SA-β-gal-positive cell numbers were counted randomly for 5 to 10 fields per slide/well. SA-β-gal-positive cells were calculated as the percentage of positive cells per area.

### Animal Study

To establish the cancer xenograft model, human colon cancer HCT116 cells were subcutaneously (s.c.) injected into the right flank of 7-week-old to 8-week-old athymic BALB/c nude mice (2 × 10^6^ /mouse) (College of Veterinary Medicine Yangzhou University, Yangzhou, China). Baicalin was given daily by i.p. injection (80 mg/kg body weight) starting on day 5 after inoculation with cancer cells for a total of 14 days. At the end of experiments, part of every tumor from different groups was collected and frozen and paraffin sectioned for senescence-associated β-galactosidase staining and immunohistochemical analyses of Ki67, p16^INK4A^ and cleaved-caspase 3, respectively. The rest of every tumor from different groups was collected and lysed for Western Blotting.

### RNA Interference

siRNA targeting DEPP and scrambled non-targeting siRNA were as follows: DEPP siRNA, CGUCACAGGAGUCUUGUGA; non-targeting siRNA, GUACGCGGAAUACUUCGAUU. The cells were transfected with specific and non-targeting siRNAs using Micropoly-transfecter Cell Reagent (Micropoly, Nanjing, China) for 24 h and then were used for subsequent experiments. The effect of RNA interference maintained at least for 1 week. In colony formation assay, cells were transfected with siRNA at the time point of 0 and 8 day.

### Statistical Analysis

All the statistical analyses were performed with Sigmaplot software (Systat Software Inc., San Jose, CA, USA). The difference between the processed samples and the normal controls was examined using Student’s *t*-test.

## Electronic supplementary material

Supplementary Figure 1

Supplementary Figure Legend

## References

[1] Rodier F, Campisi J (2011). Four faces of cellular senescence. J. Cell Biol..

[2] Roninson IB (2003). Tumor cell senescence in cancer treatment. Cancer Res..

[3] Chang BD (1999). A senescence-like phenotype distinguishes tumor cells that undergo terminal proliferation arrest after exposure to anticancer agents. Cancer Res..

[4] Gewirtz DA, Holt SE, Elmore LW (2008). Accelerated senescence: An emerging role in tumor cell response to chemotherapy and radiation. Biochem. Pharmacol..

[5] Schwarze SR, Fu VX, Desotelle JA, Kenowski ML, Jarrard DF (2005). The identification of senescence-specific genes during the induction of senescence in prostate cancer cells. Neoplasia.

[6] Kuilman T, Michaloglou C, Mooi WJ, Peeper DS (2010). The essence of senescence. Genes Dev..

[7] Robles SJ, Adami GR (1998). Agents that cause DNA double strand breaks lead to p16^INK4A^ enrichment and the premature senescence of normal fibroblasts. Oncogene.

[8] Acosta JC (2008). Chemokine signaling via the CXCR2 receptor reinforces senescence. Cell.

[9] Coppé JP (2010). A human-like senescence-associated secretory phenotype is conserved in mouse cells dependent on physiological oxygen. PLoS ONE.

[10] Koshiji M (2004). HIF-1alpha induces cell cycle arrest by functionally counteracting Myc. EMBO J..

[11] Gardner LB (2001). Hypoxia inhibits G1/S transition through regulation of p27 expression. J. Biol. Chem..

[12] Ewald JA, Desotelle JA, Wilding G, Jarrard DF (2010). Therapy-induced senescence in cancer. J. Natl Cancer Inst..

[13] Lin TY (2012). Loss of the candidate tumor suppressor BTG3 triggers acute cellular senescence via the ERK-JMJD3-p16 (INK4A) signaling axis. Oncogene.

[14] Collado M, Serrano M (2010). Senescence in tumours: evidence from mice and humans. Nat. Rev. Cancer.

[15] Melillo G (2007). Targeting hypoxia cell signaling for cancer therapy. Cancer Metastas. Rev..

[16] Manoochehri Khoshinani H, Afshar S, Najafi R (2016). Hypoxia: a double-edged sword in cancer therapy. Cancer Invest..

[17] Wilson WR, Hay MP (2011). Targeting hypoxia in cancer therapy. Nat. Rev. Cancer.

[18] Eric B (2007). The Qo site of the mitochondrial complex III is required for the transduction of hypoxic signaling via reactive oxygen species production. J. Cell Biol..

[19] Welford SM, Giaccia AJ (2011). Hypoxia and Senescence: The impact of oxygenation on tumor suppression. Mol. Cancer Res..

[20] Ragel BT, Couldwell WT, Gillespie DL, Jensen RL (2007). Identification of hypoxia-induced genes in a malignant glioma cell line (U-251) by cDNA microarray analysis. Neurosurg. Rev..

[21] Watanabe H (2005). A novel protein Depp, which is induced by progesterone in human endometrial stromal cells activates Elk-1 transcription factor. Mol. Hum. Reprod..

[22] Wang CZ (2015). Colon cancer chemopreventive effects of baicalein, an active enteric microbiome metabolite from baicalin. Int. J. Oncol..

[23] Du G (2010). Baicalin suppresses lung carcinoma and lung metastasis by SOD mimic and HIF-1α inhibition. Eur. J. Pharmacol..

[24] Yu Y, Pei M, Li L (2015). Baicalin induces apoptosis in hepatic cancer cells *in vitro* and suppresses tumor growth *in vivo*. Int. J. Clin. Exp. Med..

[25] Collado M, Blasco MA, Serrano M (2007). Cellular senescence in cancer and aging. Cell.

[26] Serrano M, Lin AW, McCurrach ME, Beach D, Lowe SW (1997). Oncogenic ras provokes premature cell senescence associated with accumulation of p53 and p16^INK4A^. Cell.

[27] Chong-Zhi W (2015). Colon cancer chemopreventive effects of baicalein, an active enteric microbiome metabolite from baicalin. Int. J. Oncol..

[28] Bonner T (1984). The human homologs of the raf (mil) oncogene are located on human chromosomes 3 and 4. Science.

[29] Bos JL (1989). Ras oncogenes in human cancer: a review. Cancer Res..

[30] Melillo G (2007). Targeting hypoxia cell signaling for cancer therapy. Cancer Metastas. Rev..

[31] Goda N (2003). Hypoxia-inducible factor 1alpha is essential for cell cycle arrest during hypoxia. Mol. Cell. Biol..

[32] Deng Q, Liao R, Wu BL, Sun P (2004). High Intensity ras Signaling Induces Premature Senescence by Activating p38 Pathway in Primary Human Fibroblasts. J. Biol. Chem..

[33] Zhu B (2014). PPARβ_δ promotes HRAS-induced senescence and tumor suppression by potentiating p-ERK and repressing p-AKT signaling. Oncogene.

[34] Choi SH (2013). Regulation of ROS-independent ERK signaling rescues replicative cellular senescence in ex vivo expanded human c-kit-positive cardiac progenitor cells. Int. J. Cardiol..

[35] Wang Z (2013). N terminus of ASPP2 binds to Ras and enhances Ras-Raf-MEK-ERK activation to promote oncogene-induced senescence. Proc. Natl Acad. Sci. USA.

[36] Barzegar A, Moosavi-Movahedi AA (2011). Intracellular ROS protection efficiency and free radical-scavenging activity of curcumin. PLoS ONE.

[37] Singh SV (2005). Sulforaphane-induced cell death in human prostate cancer cells is initiated by reactive oxygen species. J. Biol. Chem..

