# Supplementary material for: Baicalin induces cellular senescence in human colon cancer cells via upregulation of DEPP and the activation of Ras/Raf/MEK/ERK signaling
Source: Cell Death Dis. 2018 Feb 13;9(2):217. doi: 10.1038/s41419-017-0223-0 (PMC5833439; doi:10.1038/s41419-017-0223-0)
Supplement: Supplementary file 2 — Supplementary Figure Legend [file 41419_2017_223_MOESM2_ESM.docx]

**Supplementary Figure Legend**

**Supplementary Figure 1:**  Baicalin induced senescence in colon cancer cells *in vivo*. Tumor sections were subjected to SA-β-Gal staining and IHC for Ki-67, p16^INK4A^ and cleaved-caspase 3. Original magnification was 200×.
